# Evaluation of Postoperative Pain and Apical Outcomes Following Three Distinct Obturation Techniques: A Randomized Controlled Trial

**DOI:** 10.7759/cureus.107873

**Published:** 2026-04-28

**Authors:** Pragati Agarwal, Pravin Kumar, Vinay Kumar Chugh, Sharmila Shanmugam, Soundharrajan P, Karishma Pathak

**Affiliations:** 1 Conservative Dentistry and Endodontics, All India Institute of Medical Sciences, Jodhpur, Jodhpur, IND; 2 Orthodontics and Dentofacial Orthopedics, All India Institute of Medical Sciences, Jodhpur, Jodhpur, IND

**Keywords:** apical extrusion, apical seal, obturation, postoperative pain, root canal treatment

## Abstract

Background and aim

Postoperative pain following root canal treatment (RCT) can be influenced by the obturation technique, as variations in apical sealing and material extrusion may affect periapical tissue response. The aim of this study was to evaluate postoperative pain and apical outcomes following three different obturation techniques in patients diagnosed with symptomatic irreversible pulpitis, with or without apical periodontitis.

Methods

Seventy-five single-rooted teeth requiring RCT were randomly allocated into three groups based on the obturation technique used: cold lateral condensation, warm vertical compaction, and carrier-based obturation (CBO). Baseline pain was recorded using a Visual Analogue Scale (VAS). All procedures were performed by a single experienced endodontist in two visits. Postoperative pain levels were recorded using the VAS at six, 24, 48, and 72 hours. The incidence of apical sealing of lateral canals and ramifications, as well as obturation material extrusion, was evaluated radiographically.

Results

Intergroup comparison of postoperative pain scores using the Kruskal-Wallis H test showed no statistically significant difference among the three obturation techniques at any of the evaluated time intervals (p > 0.05). The CBO group demonstrated a higher incidence of sealing of apical ramifications and lateral canals; however, the overall difference among groups was not statistically significant (p = 0.056). A significantly higher incidence of obturation material extrusion was observed in the CBO group (p = 0.020).

Conclusions

The obturation technique did not significantly affect postoperative pain. The CBO method demonstrated a higher incidence of apical sealing but was associated with increased material extrusion. The selection of the obturation technique should balance sealing efficacy with the risk of extrusion.

## Introduction

Endodontic therapy aims to achieve effective chemo-mechanical preparation (CMP) of the root canal to eliminate pulpal and periradicular microorganisms [1]. Microbial reduction is accomplished through a combination of mechanical instrumentation and chemical disinfection, followed by obturation to create a fluid-tight seal using gutta-percha and an endodontic sealer [2,3].

Various obturation techniques, including cold lateral condensation (CLC), warm vertical compaction (WVC), and thermoplasticized methods, have been used to improve canal sealing [4]. CLC remains a commonly used obturation technique due to its predictability, cost-effectiveness, and operator control [5]. However, it may result in voids and inadequate adaptation in complex canal anatomies [6]. In contrast, contemporary thermoplasticized techniques, including WVC and carrier-based obturation (CBO), are increasingly utilized due to their potential to improve gutta-percha flow, canal adaptation, and overall filling quality [7-9].

Postoperative pain is a common outcome following endodontic treatment, with reported prevalence ranging from 2.5% to over 60%. Pain typically develops within six to 12 hours after treatment, peaks at approximately 24 hours in nearly 40% of patients, and gradually decreases to around 11% after one week [10]. It is multifactorial and may be influenced by pulpal status, treatment variables, and periradicular tissue irritation, including extrusion of obturation materials beyond the apical foramen [11].

In addition to patient comfort, the quality of apical obturation, particularly the filling of apical ramifications and lateral canals, is a critical determinant of long-term treatment success. Inadequate filling of these anatomical complexities can result in residual microbial presence, compromised periapical healing, and increased risk of posttreatment disease [12].

Despite the clinical importance of postoperative pain and apical sealing, few randomized controlled trials have directly compared commonly practiced obturation techniques regarding both outcomes. Existing evidence largely derives from in vitro studies, providing limited guidance for clinical decision-making. This randomized controlled trial compared CLC, WVC, and CBO in teeth diagnosed with symptomatic irreversible pulpitis, with or without apical periodontitis. The study assessed the incidence and intensity of postoperative pain at multiple time intervals and the incidence of effective sealing of apical canal complexities and obturation material extrusion. The null hypothesis proposed no significant differences among the three obturation techniques in postoperative pain or apical outcomes following root canal treatment (RCT).

## Materials and methods

Study design and sample size calculation

Patients for this parallel-arm randomized controlled trial were recruited from the outpatient department between October 2024 and September 2025. Ethical approval was obtained from the Institutional Ethics Committee (approval AIIMS/IEC/2024/5078), and the trial was registered with the Clinical Trials Registry - India (CTRI/2024/06/068236).

The sample size was calculated based on the comparison of two independent proportions (incidence of postoperative pain at six hours) between the study groups. Based on previous literature [13], the expected proportion of patients experiencing pain was assumed to be 70% in the CLC group and approximately 99% in the CBO group, yielding an effect size of 0.96.

Assuming a significance level of 5% (α = 0.05) and a power of 80%, the minimum required sample size was calculated to be 23 participants per group. To compensate for possible attrition, the sample size was increased to 25 participants per group, resulting in a total sample size of 75 participants. Written informed consent was obtained from all participants.

Inclusion and exclusion criteria

Healthy patients without systemic diseases or allergic reactions (American Society of Anesthesiologists (ASA) I and ASA II), aged between 18 and 65 years, with single-rooted teeth having a single apical exit, fully formed apices, and no prior RCT, and with periodontally healthy teeth diagnosed with symptomatic irreversible pulpitis or apical periodontitis, were included in the study. Patients who had taken analgesics or anti-inflammatory drugs within 12 hours prior to root canal obturation, pregnant or lactating women, and teeth showing radiographic evidence of resorption (internal/external), root fracture, calcification, or advanced bony destruction, as well as teeth with previously initiated or completed RCT, were excluded.

Randomization and allocation concealment

**Figure 1:**
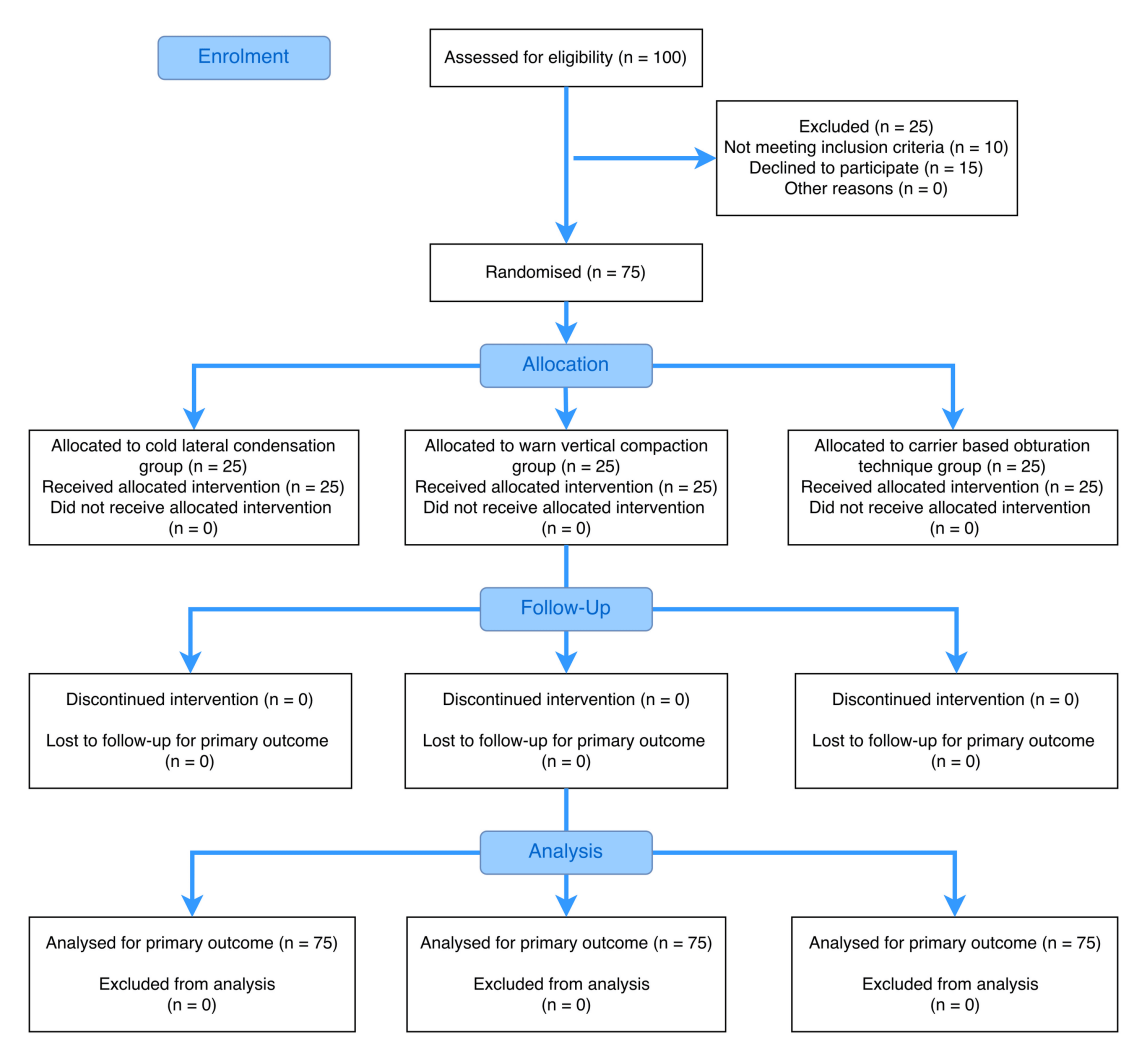
CONSORT flow diagram illustrating participant flow through each stage of the randomized controlled trial CONSORT, CONsolidated Standards of Reporting Trials

Block randomization with a 1:1:1 allocation ratio was performed using an online randomization tool by central library personnel. Participants were sequentially allocated to three groups as per the CONsolidated Standards of Reporting Trials (CONSORT) guidelines (Figure 1). Allocation concealment was ensured using sealed, opaque, sequentially numbered envelopes. A double-blind technique was used, with participants and outcome assessors blinded to group allocation; operator blinding was not feasible.

Clinical procedures (RCT protocol)

Local anesthesia was administered using 2% lidocaine with 1:80,000 epinephrine, followed by rubber dam isolation and access cavity preparation. Canal patency was established with a #10 stainless steel K-file. Working length was determined using an electronic apex locator (CanalPro Apex Locator Compact, Coltene, Langenau, Germany) and confirmed radiographically. Canal preparation was performed using hand ProTaper^®^ Universal files with Glyde as a lubricant and irrigation with 3% sodium hypochlorite and saline. A hand instrumentation approach was preferred to allow greater tactile control and standardization of canal preparation, thereby minimizing procedural variability and reducing the risk of instrument separation and other procedural errors.

At the second visit, canals were irrigated with 5 mL of 3% sodium hypochlorite, followed by 2 mL of 17% EDTA for one minute and a final saline rinse. Canals were dried with sterile paper points, and a zinc oxide-eugenol sealer, Endoseal (Prevest DenPro Limited, Jammu, India), was applied using a lentulospiral. All procedures were performed under 3.5× magnification with LED illumination.

**Figure 2:**
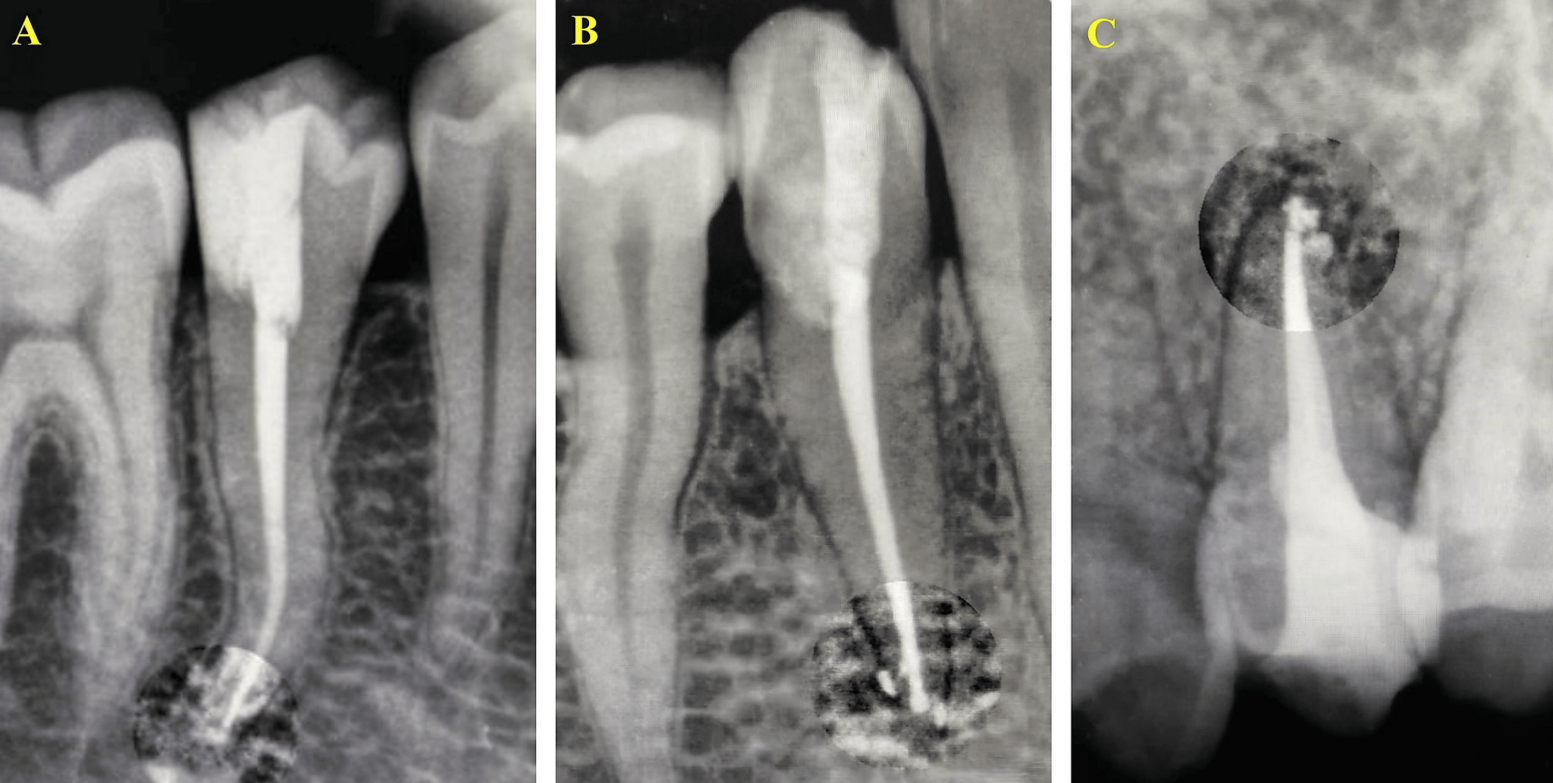
Representative intraoral periapical radiographs showing obturation in the three study groups (A) CLC. (B) WVC. (C) CBO. CBO, carrier-based obturation; CLC, cold lateral condensation; WVC, warm vertical compaction

Group 1 (CLC, n = 25) canals were obturated by fitting a master gutta-percha cone to working length with confirmed tug-back, followed by lateral condensation using a finger spreader and accessory cones until no further penetration was possible (Figure 2A). In Group 2 (WVC; GuttaSmart™, Dentsply Sirona, Ballaigues, Switzerland; n = 25), a master cone was positioned 0.5-2 mm short of working length, and after sealer application, vertical compaction was performed with a heated GuttaSmart plugger to create a 4-5 mm apical plug, followed by backfilling with thermoplasticized gutta-percha in 3 mm increments (Figure 2B). Group 3 (CBO; GuttaCore™, Dentsply Sirona; n = 25) canals were obturated by heating the GuttaCore obturator according to the manufacturer’s instructions and inserting it to working length, after which the carrier shaft was severed at the canal orifice (Figure 2C).

Postoperative assessment and outcome measures

Post-endodontic restoration was completed with composite resin. Patients were prescribed an anti-inflammatory analgesic (ibuprofen 400 mg) and instructed to take it only in case of severe postoperative pain, with a maximum frequency of every six to eight hours as needed. Postoperative pain was assessed using a Visual Analogue Scale (VAS) at six, 24, 48, and 72 hours. Analgesic consumption was documented alongside VAS scores to allow contextual interpretation of postoperative pain outcomes. No modification of VAS scores was performed based on analgesic intake. An independent blinded endodontist evaluated postoperative radiographs for apical canal filling and material extrusion.

Statistical analysis

Data were entered and analyzed using IBM SPSS Statistics for Windows, version 25.0 (released 2017; IBM Corp., Armonk, NY, USA). Statistical comparisons for gender, apical sealing, and apical extrusion were performed using the chi-square test or Fisher’s exact test, as appropriate. Age and VAS pain scores were expressed as mean ± SD or median with IQR. Data normality was assessed using the Shapiro-Wilk test. As the data were non-normally distributed, intergroup comparisons were carried out using the Kruskal-Wallis test. Post hoc pairwise comparisons were performed using Bonferroni correction to adjust for multiple comparisons (Table 1). Within-group comparisons over time were analyzed using the Friedman test. A p-value ≤ 0.05 was considered statistically significant.

**Table 1:** Post hoc pairwise comparisons of VAS scores at different time points with Bonferroni correction While comparing VAS scores at different time points, no significant intergroup differences were observed in post hoc pairwise analysis with Bonferroni correction (p > 0.05). VAS, Visual Analogue Scale

p-Values of post hoc analysis	Group 1 vs Group 2	Group 1 vs Group 3	Group 2 vs Group 3
Preoperative VAS	0.256	1.000	1.000
VAS at six hours	1.000	1.000	0.465
VAS at 24 hours	1.000	1.000	1.000
VAS at 48 hours	1.000	1.000	0.579
VAS at 72 hours	1.000	0.580	0.210

## Results

No statistically significant differences were observed among the groups with respect to demographic variables, including age and gender (p > 0.05). Preoperative VAS scores were comparable across the three groups (p > 0.05). Postoperative VAS scores at six, 24, 48, and 72 hours did not differ significantly between groups (p > 0.05) (Table 2). Although Group 2 (WVC) consistently demonstrated numerically lower pain scores and Group 3 (CBO) showed relatively higher values at various time points, these differences were not statistically significant.

**Table 2:** VAS score and median of the three groups at different time intervals ^*^ Kruskal-Wallis H test ^†^ Friedman test p < 0.05 = statistically significant VAS, Visual Analogue Scale

Variable	Group 1	Group 2	Group 3	p-Value^*^
Preoperative VAS				0.245
Median (IQR)	80 (50)	50 (60)	80 (40)	
VAS at six hours				0.137
Median (IQR)	0 (0)	0 (0)	0 (10)	
VAS at 24 hours				0.546
Median (IQR)	0 (0)	0 (0)	0 (0)	
VAS at 48 hours				0.560
Median (IQR)	0 (0)	0 (0)	0 (0)	
VAS at 72 hours				0.158
Median (IQR)	0 (0)	0 (0)	0 (0)	
p-Value^†^	<0.001	<0.001	<0.001	

Rescue analgesic consumption was minimal, with three patients in the CBO group, two patients in the WVC group, and one patient in the CLC group requiring analgesics within the first 24 hours postoperatively. For apical sealing, filling of apical canal complexities was observed in Group 1 (CLC; n = 8, 32%) and Group 2 (WVC; n = 5, 20%), with no statistically significant difference between the two groups (p = 0.333). Similarly, no significant difference was noted between Group 1 (CLC; n = 8, 32%) and Group 3 (CBO; n = 13, 52%) (p = 0.152). However, a statistically significant difference was observed between Group 2 (WVC; n = 5, 20%) and Group 3 (CBO; n = 13, 52%) (p = 0.018), with the CBO group demonstrating superior filling of apical canal complexities (Table 3, Table 4).

**Table 3:** Comparison of apical sealing and extrusion between groups ^*^ Chi-square test ^†^ Fisher’s exact test p < 0.05 = statistically significant

Outcomes	Status	Group 1, n (%)	Group 2, n (%)	Group 3, n (%)	p-Value
Apical sealing	Present	8 (32)	5 (20)	13 (52)	0.056^*^
	Absent	17 (68)	20 (80)	12 (48)	
Extrusion	Present	3 (12)	7 (28)	12 (48)	0.020^†^
	Absent	22 (88)	18 (72)	13 (52)	

**Table 4:** Intergroup pairwise analysis of apical sealing and extrusion (p-values) ^*^ Fisher’s exact test ^†^ Chi-square test p < 0.05 = statistically significant

Outcomes	Group 1 vs Group 2	Group 1 vs Group 3	Group 2 vs Group 3
Apical sealing	0.333^†^	0.152^†^	0.018^†^
Extrusion	0.157^*^	0.005^*^	0.145^†^

Regarding apical extrusion, Group 3 (CBO; n = 12, 48%) demonstrated a significantly higher incidence compared with Group 1 (CLC; n = 3, 12%) (p = 0.005). Extrusion was also observed in Group 2 (WVC; n = 7, 28%); however, differences between Group 2 and the other groups were not statistically significant (Table 3, Table 4).

## Discussion

Posttreatment pain remains one of the most frequently reported concerns in endodontics, despite advances in rotary instrumentation, irrigation protocols, and obturation techniques [14]. Effective prevention and management of postoperative discomfort are therefore critical components of successful root canal therapy [15].

While earlier studies have reported no significant difference in postoperative complications between single- and multi-visit endodontic treatment, this study employed a two-visit protocol to minimize potential confounding factors related to CMP [16]. CMP is known to independently contribute to postoperative pain due to mechanical instrumentation and irrigation-related factors. Therefore, obturation was deferred to a subsequent visit to minimize the influence of CMP-related pain on the outcome assessment. This approach allowed a clearer evaluation of pain attributable primarily to the obturation procedure.

A ZOE-based sealer was used uniformly across all groups to standardize obturation. ZOE sealers have garnered significant attention due to their antimicrobial properties, primarily attributed to the release of eugenol [17]. The use of a single sealer type ensured that differences in postoperative outcomes could be attributed to the obturation technique rather than sealer variability.

Although previous studies have suggested that preoperative pain intensity may influence postoperative outcomes [18], no statistically significant differences were observed in baseline pain levels among the groups in the present trial. This indicates that preoperative pain did not confound the assessment of postoperative discomfort. Similarly, no statistically significant differences were observed among the groups in postoperative VAS scores. Although the CBO group demonstrated numerically higher mean pain scores at six, 24, 48, and 72 hours, these differences were not statistically significant. This numerical variation may be considered exploratory and could be further investigated in relation to procedural factors such as potential material extrusion; however, no causal relationship can be established based on the present findings. The relationship between apical extrusion and postoperative discomfort has been well-documented, with greater extrusion often correlating with higher pain levels [19,20]. However, in the present study, such an association could not be confirmed due to the lack of statistically significant intergroup differences.

The results of the present study showed a 48% incidence of extrusion of the obturation material in the CBO group. Carrier-based systems, such as Gutta-Core, utilize a central cross-linked gutta-percha carrier surrounded by flowable thermoplasticized gutta-percha [21]. While this configuration allows better adaptation to canal irregularities, it may compromise apical length control during insertion, contributing to the higher extrusion rates observed in this group [22]. These findings are consistent with previous studies reporting higher rates of extrusion with carrier-based techniques such as Thermafil, which is thought to result from the rigid core delivering gutta-percha more forcefully into the canal, increasing the risk of apical extrusion [23].

At 72 hours, the lowest mean postoperative pain was recorded in the WVC group, while the CLC group showed slightly higher pain scores than WVC. The incidence of over-extrusion was 12% in the CLC group and 28% in the WVC group. The minimal extrusion observed in the CLC group may be explained by its superior apical length control. Overall, the results suggest that although all three obturation techniques provide comparable postoperative comfort, carrier-based systems may be associated with higher early postoperative pain due to their greater tendency for material extrusion [8].

Effective sealing of apical ramifications and lateral canals is a key objective of root canal obturation, as it entraps residual microorganisms in otherwise inaccessible areas, depriving them of space and essential nutrients, thereby supporting periapical health [14]. In the present study, Gutta-Core provided superior filling of apical ramifications (52%), significantly outperforming CLC (32%) and WVC (20%). This enhanced performance can be attributed to the use of heated gutta-percha in carrier-based systems, which exhibits thixotropic behavior, reducing viscosity and enhancing flow into lateral canals, isthmuses, and apical irregularities. These findings align with Al-kattea et al. [24], who also reported improved adaptation of carrier-based gutta-percha in complex canal anatomies. Overall, the results highlight that the obturation technique plays a critical role in achieving effective apical sealing, and techniques that combine heat and controlled delivery of gutta-percha are more capable of filling complex apical anatomy than traditional lateral condensation alone.

The strengths of this trial include strict inclusion criteria and blinded outcome assessment to minimize bias. The use of standardized instrumentation and a single sealer ensured that results reflected differences in obturation techniques rather than material-related variability. Postoperative pain was measured at multiple intervals using a validated scale. However, the study was limited to single-rooted teeth, which may restrict the generalizability of the findings to multi-rooted or anatomically complex teeth. Radiographic evaluation was performed using two-dimensional periapical radiographs, which are limited in detecting lateral canals and apical ramifications due to the lack of 3D detail. Although cone-beam CT provides superior visualization, its routine use for postoperative obturation assessment is not justified due to higher radiation exposure, cost, and ethical concerns; therefore, periapical radiography was used, as it remains the standard method in clinical practice. Additionally, postoperative pain was self-reported, rendering it susceptible to individual variations in pain perception and compliance with VAS recording. Furthermore, although the outcome assessor was blinded, inherent radiographic differences between obturation techniques may have allowed partial identification of the intervention, potentially introducing assessment bias. Future studies should include multi-rooted and anatomically complex teeth to better evaluate the clinical performance of different obturation techniques in diverse canal systems.

## Conclusions

All three obturation techniques resulted in comparable postoperative pain, with no statistically significant differences observed between groups. Although minor variations were noted, these findings do not indicate a clinically meaningful difference in pain outcomes. While the CBO system achieved superior filling of apical canal anatomy, it was also associated with increased apical extrusion. CLC provided better apical control, whereas WVC offered a balance between sealing ability and apical control. Clinical considerations, including canal anatomy and the relative importance of apical control and 3D filling, should guide the choice of obturation technique.
